# Callerya Atropurpurea shells derived nitrogen doped carbon quantum dots of electrodes for symmetrical and asymmetrical supercapacitors

**DOI:** 10.1038/s41598-025-15098-2

**Published:** 2025-08-18

**Authors:** Abdulrahman Oyekanmi Adeleke, Mohd Arif Dar, Temitope T. Dele-Afolabi, R. C. Omar, Rasykin Roslan, Akil Ahmad, Ebrahim Mahmoudi, Chua Siew Fen, Ali Orozi Sougui, Mohammed B. Alshammari

**Affiliations:** 1 Institute of Energy Infrastructure (IEI), Universiti Tenaga Nasional (UNITEN), Jalan IKRAM-UNITEN, Putrajaya Campus, Kajang, 43000 Selangor Malaysia; 2 Department of Physics, Centre for Ionics Universiti Malaya, Universiti Malaya, Kuala Lumpur, 50603 Malaysia; 3 Faculty of Allied Health Sciences, Chettinad Hospital, and Research Institute, Chettinad Academy of Research and Education, Kelambakkam, 603103 Tamil Nadu India; 4 Institute of Power Engineering (IPE), Universiti Tenaga Nasional (UNITEN), Jalan IKRAM-UNITEN, Putrajaya Campus, Kajang, 43000 Selangor Malaysia; 5 Chemistry Department, College of Sciences and Humanities, Prince Sattam bin Abdulaziz University, P.O. Box 83, Al-Kharij, 11942 Saudi Arabia; 6 Department of Chemical and Process Engineering, Universiti Kebangsaan Malaysia, Bangi, 43600 Selangor Malaysia; 7 Université de N’Djamena, 1117 N’Djamena, Chad

**Keywords:** *Callerya Atropurpurea* shells, Activated carbon, Carbon quantum dots, Supercapacitors, Asymmetrical, Symmetrical and energy storage, Energy storage, Renewable energy, Energy science and technology, Engineering, Materials science

## Abstract

*Callerya Atropurpurea* shells were utilized as activated carbon precursors in a one-stage activation process at 700 °C using H_2_SO_4_, NaOH and KOH as activating agents. Herein, carbon quantum dots (CQDs) were produced via self-doping using urea as a nitrogen source. The structural, functional, and morphological properties of the doped active materials were examined using X-ray diffraction, Fourier transform infrared spectroscopy, and Raman spectroscopy. The elemental composition was conducted using energy-dispersive X-ray spectroscopy, and surface sensitivity was determined using X-ray photoelectron spectroscopy techniques. The surface properties showed that the nitrogen-doped CQDs produced good crystallinity with an abundance of nitrogen heteroatoms attached to the surface, facilitating the conductivity of the devices. The electrodes of NCQDs-1, NCQDs-2 and NCQDs-3 were prepared and used for the fabrication of asymmetric and symmetric supercapacitor electrodes. The NCQDs-3 electrode used in the asymmetric and symmetrical devices showed a higher specific capacitance of 22 F/g at a current density of 0.5 A/g. Also, the NCQDs-3 electrode achieved the highest coulombic efficiency of 98% and a capacitive retention of 99% even after 1000 GCD cycles.

## 1. Introduction

In the last few decades, over the passage of time, energy utilization, fossil fuel energy consumption, CO_2_ emission and other associated pollution, such as climate change and global warming, have caused a standard shift and remarkable attention towards creating low-cost and eco-friendly energy storage devices^1,2^. Electrochemical energy storage systems are increasingly becoming pivotal to the sustainability of the field of new energy^3–6^. Among different sources of energy storage applications, supercapacitors are known to possess safe operational processing, prolonged life cycle and quick charge-discharge capacities compared to the utilization of rechargeable batteries^7–10^. However, improving the inherent low energy density by identifying the suitability of electrolytes and active materials for the fabrication of the electrodes, which guarantees higher electrochemical performance, requires a research focus that can best inspire innovative solutions to the existing energy applications^11–13^. Recently, the utilization of supercapacitors derived from carbonaceous source active materials as electrodes to entrench green technology has generated remarkable interest, due to high electrical conductivity, which enables intrinsic high power density, quick and effective charge-discharge, that guarantees long life cycle stability with higher capacitance compared to conventional capacitors^14^.

Supercapacitors derived from carbon materials generally demonstrate electric double-layer capacitor (EDLC-type) storage characteristics with enhanced ion adsorption-desorption kinetics, which mainly depend on their structural composition^15^. Commercially derived EDLCs from activated carbons under different activation conditions have been found to exhibit a capacitance between 17 F/g to 20 F/g^16^. However, to achieve suitable electrochemical performance remains a challenge in the production of large-scale commercial-like electrode materials^17–20^. For instance, Farzana et al.^21^ produced a supercapacitor using activated carbon derived from compact discs and achieved a capacitance of 31 F/g, although using 50 mV/s scan rate, and 80% of cycle stability for up to 800 cycles was achieved. Chen et al.^22^ produced activated carbon paper as self-supporting electrodes and achieved a capacitance of 14.5 F/g to 21.6 F/g which was achieved between 0.35 and 10 mA/cm^2^ current densities. Elyazed et al.^23^ fabricated a facile and efficient electrode using SnO_2_/activated carbon waste for supercapacitor applications. The calcined activated carbon wastes achieved specific capacitance of 1.42 F/g. Meanwhile, improved capacitance was achieved with the incorporation of SnO_2_ (30.46 F/g).

Agricultural wastes can be utilized as a source material for various applications^24–26^. Recently, researchers have expanded the utilization of carbonaceous materials using quasi-spherical carbon nanoparticles, known as carbon quantum dots (CQDs)^27^, as a precursor material from agricultural biomasses for energy storage applications^28^. These CQDs are attributed to their excellent electrical properties, easy functionalization, environmental friendliness and high chemical stability^29,30^. The utilization of waste biomass-derived CQDs as a source of active materials for the fabrication of electrode devices is crucial and significant for the improvement of the capacitance and the storage properties of supercapacitors^31^. Several studies have enhanced the electrochemical performances of porous carbon by doping heteroatoms within the carbon structure to facilitate a synergistic interaction between the atoms to provide sufficient pathways and interspaces for effective ion transfer as an effective agent to improve the conductivity and electrochemical performance^32–37^. In practice, the synthesis process of doping heteroatoms is usually via multidimensional routes, and the processes are costly, thereby creating a barrier^2,38–41^. Urea as a doping source is more preferable to the use of ammonia, melamine and some other doping sources due to the ability of its structure during synthesis to facilitate effective incorporation of nitrogen into the carbon lattice^42^. Moreover, under hydrothermal pyrolytic conditions, the decomposition of urea releases nitrogenous compounds such as, amines and nitriles, which can be easily incorporated in the CQDs^43^. The arrays of functional groups, such as amide, amine and carboxyl groups present in urea, improve the surface chemistry and functional properties of CQDs. For instance, amide groups in urea provide active sites for functionalization through covalent bonding^42–44^. Urea is primarily utilized as a fertilizer is a widely available and inexpensive chemical, implying that the low cost of urea is very beneficial for the production of CQDs, making it as a very significant doping source for research on CQDs, thereby expanding the frontiers of their potential applications^45^. Hydrothermal and microwave-assisted synthesis routes of CQDs using urea are environmentally friendly due to minimal requirement for harsh chemicals or energy consumption^46^.

Pallavolu et al.^47^ synthesized nitrogen-doped graphitic carbon sheets and could achieve 55.7 F/g of specific capacitance at 1 A/g. The symmetric electrode achieved capacity retention of 92.5% through 10,000 cycling tests. Sung et al.^48^ fabricated co-doped carbon quantum dots (NCQDs) using sulfur and nitrogen on foil from copper for lithium-based capacitors. The NCQD/Cu produced 11.9 F/g capacitance and a 76.1% cycle stability. Aramid nanofiber-derived nitrogen-doped aerogel carbon was fabricated by Gao et al.^49^. The device achieved a capacitance of 32 F/g and also 72% retention capacity through a 1200-cycle stability test. The electrochemical behaviour of rGO/GQDs doped Fe-MOF nanocomposites was investigated. This device, which was fabricated using a one-stage hydrothermal synthesis, achieved a capacitance of 54.2 F/g and 95% capacity retention after 5000 cycles. The utilization of agricultural wastes as precursors for the synthesis of doping materials can further expand the frontiers of energy storage applications^50^. Baslak et al.^51^ developed a green synthesis of CQDs from Sideritis vuralii. It was revealed that the electrode demonstrated a rechargeable symmetrical capacitor behaviour with a specific capacitance of 10.42 F/g. Nitrogen-doped carbon capsule (NC) CoMn_2_O_4_ nanoflower electrode material was fabricated by Guo et al.^52^. The electrochemical behaviour of the device achieved a specific capacitance of 15.85 F/g, with a capacity retention of 92.1%. Chen et al.^53^ developed a Zeolitic imidazolate framework-67 (ZIF67) N-doped carbon composite as a supercapacitor device. The fabricated electrode achieved a specific capacitance 2 F/g at 100% retention capacity from the cycling test. Therefore, doping CQDs from agricultural waste biomasses as precursors offers a compelling pathway towards achieving environmentally friendly, high-performance supercapacitors with remarkable stability. Thus, advancing sustainable energy storage technologies.

Hydrothermal synthesis techniques have comparative advantages over other methods due to the ability to generate high-quality and environmentally friendly carbonaceous materials from waste precursors^54^. Driven by environmental considerations, the utilization of toxic and harmful chemicals for the preparation of CQDs has necessitated curiosity in the use of spent agricultural biomasses, which are readily available as a valuable precursor material. The synthesis of spent agricultural wastes via the hydrothermal route offers sustainable pathways towards increasing the yield and large-scale production of nitrogen-doped CQDs^55^. The hydrothermal technique offers control over the functional properties of the precursor by ensuring tunability of reaction parameters such as pressure, temperature, and the residence time^13^. Due to the ability of hydrothermal processing to facilitate rapid heating and quenching cycles, carbonization and activation of the carbon precursors can be achieved in a controlled manner, thereby enabling uninhibited production without the variability of batch-to-batch processes^56^. In the hydrothermal synthesis of waste precursor, controlling parameters such as the structure and chemical composition of the starting material are essential for the determination of the yield of the carbon, and also reflect on the doping and conductivity of the produced carbon^57–59^. A highly homogenous and improved conductive surface is likely to be derived with reduced sizes of the starting materials^60^. Additionally, higher temperatures favour improved carbonization, pore size distribution, and conductivity^61^. Also, the influence of longer reaction time promotes complete carbonization and pore sizes^62^. The influence of these parameters on the end product of the hydrothermal synthesis. While a higher solid-liquid ratio increases the carbon yield, a lower solid-liquid ratio impacts improved dispersion and the likely formation of homogeneous end products^63^.

Herein, entrenching energy storage by adding to the literature, we have reported a facile hydrothermal synthesis strategy for nitrogen-doped carbon quantum dots (NCQDs) using *Callerya Atropurpurea* shells are easily sourced biomass having the potential for larger-scale, wide applications, especially for the production of carbon-based precursors. The abundance of these shell components makes them ideal precursors for thermochemical conversion processes such as pyrolysis. The pyrolysis of the shells of *Callerya Atropurpurea* provides the essential component for the formation of carbonaceous materials after the materials are subjected to different chemical routes, which implies that these materials provide suitable sites for heteroatom doping on their surface^64^. Moreover, Callerya atropurpurea shells are readily available agricultural wastes, can be processed into CQDs due to their abundant functional groups, which include the hydroxyl (-OH), carbonyl (C = O), and carboxyl (-COOH) groups. These groups are essential for enhancing the electrochemical behaviour of the materials and subsequently enabling their suitability for supercapacitor applications. Therefore, these wastes can facilitate energy storage and charge transfer during electrochemical reactions.

Hence, in this study, we synthesize nitrogen (N) doped on activated carbon sources derived from waste biomass of *Callerya Atropurpurea* shells to derive carbon quantum dots using H_2_SO_4,_ NaOH and KOH as activation agents, which is favourable for EDLC in a symmetric and asymmetric electrode system. The samples were code-named NCQDs-1, NCQDs-2 and NCQDs-3, respectively. The synthesized NCQDs-1, NCQDs-2 and NCQDs-3 carbon quantum dots are characterized using X-ray diffraction spectroscopy (XRD), Fourier transform infrared spectroscopy (FTIR), X-ray photoelectron spectroscopy (XPS), Raman spectroscopy, including high-resolution transmission electron microscopy (HRTEM) and elemental mapping techniques. Furthermore, the electrodes of NCQDs-1, NCQDs-2 and NCQDs-3 were fabricated and used for both symmetrical and asymmetrical device formation as supercapacitors. The supercapacitive performances of the fabricated devices were evaluated through cyclic voltammograms (CV), galvanostatic charging and discharging (GCD), electrochemical impedance spectroscopy (EIS) and cyclic stability tests.

## 2. Experimental section

### 2.1 Synthesis of CAS-AC

*Callerya Atropurpurea* shells (CAS) were sourced from agricultural farms at the Universiti Putra Malaysia (UPM), Serdang, Malaysia. The precursor was washed exhaustively using deionized (DI) water to remove surface impurities and other adhering dirt particles. The samples were pre-crushed, sieved (< 1 mm), washed, and dried in an oven at 150 °C for 2 h. In a one-stage synthesis process, 10 g of the as-prepared precursor was carbonized for 3 h using a tubular furnace at 700 °C. The carbonized waste biomasses were impregnated with 1 M H_2_SO_4_, NaOH, and KOH, at an activating agent: carbon ratio of 1.5 or 2 during 6 h under continuous stirring. Afterwards, the materials were oven-dried overnight at 110 °C. The impregnated carbons were put into the autoclave reactor, which did not exceed 1/3 of the reactor volume. The reactors were sealed, allowing the materials to be hydrothermally activated for 2 h at 300 °C. The residual concentration of the activating agent from the activated carbons was eliminated by rinsing the samples with distilled water and 0.1 M hydrochloric acid solution. The resulting AC samples were obtained after being dried at 105 °C for 12 h. The synthesized nanoparticles were named CQDs-1 (H_2_SO_4_), CQDs-2 (NaOH), and CQDs-3 (KOH), respectively.

### 2.2 Synthesis of CAS-AC/NCQDs

**Fig. 1:**
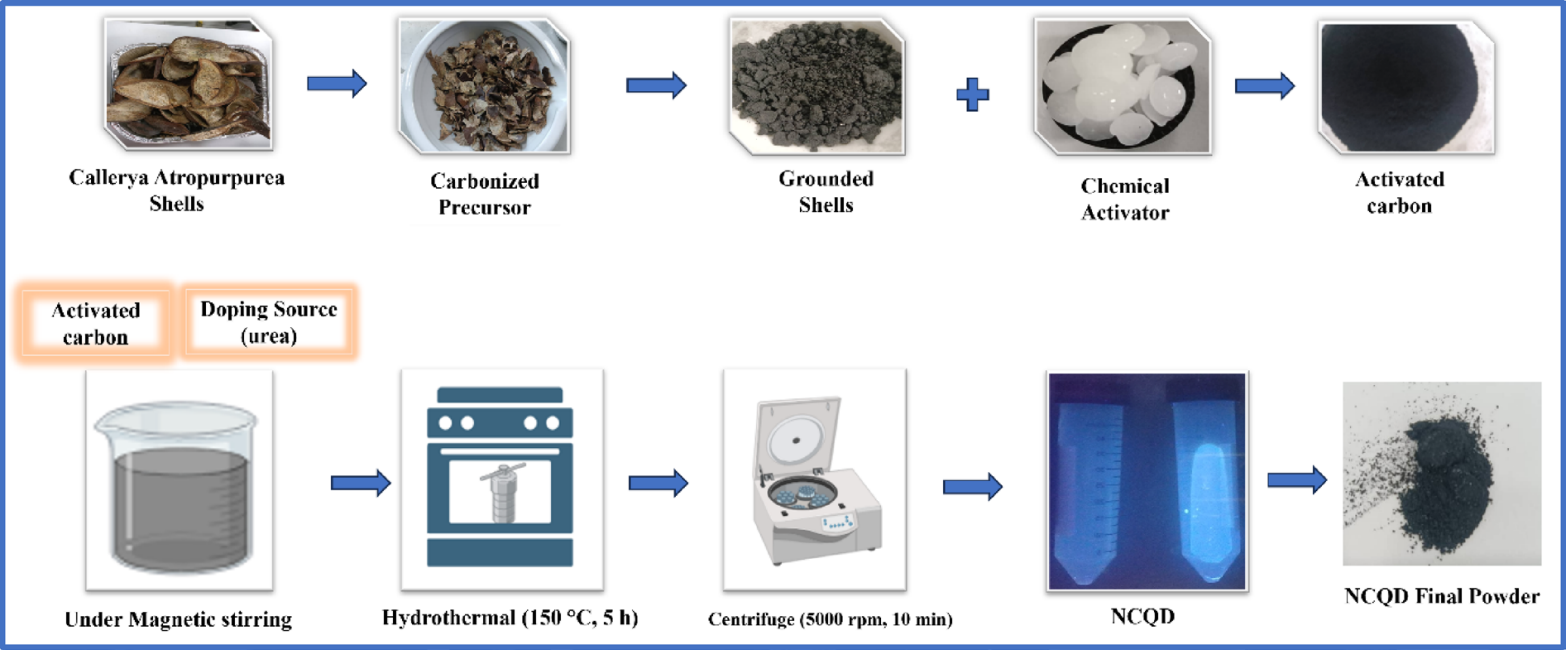
Schematic preparation of the NCQDs-1, NCQDs-2, and NCQDs-3, respectively.

The preparation and the synthesis route of NCQDs is shown in Fig. 1. Firstly, 0.18 g of urea was added to 0.21 g of CQDs separately and dispersed by ultrasonication into 10 mL DI water for 30 min. Next, the suspension was sealed in a Teflon-lined stainless-steel autoclave and kept in an oven for 5 h at 150 °C. Each sample was allowed to cool after 24 h and centrifuged for 10 min at 5000 rpm. After drying, the final products obtained were named NCQDs-1, NCQDs-2, and NCQDs-3, respectively.

### 2.3 Characterization studies

The crystallinity of the prepared NCQD samples was examined using an XRD (X’pert diffractometer). The Raman spectra were obtained in the range of 100–3000 cm^−1^ using a Lab RAM setup (model HR 800 at 633 nm). The surface morphologies of the fabricated NCQD samples were obtained using the FESEM (Zeiss Ultra 60 at 5 kV) and HRTEM (Zeiss-2100). EDS equipped with FESEM, and lastly, XPS (Thermo-Scientific 250XI) was used to obtain the chemical composition of samples.

### 2.4 Fabrication of electrodes

The NCQDs-1, NCQDs-2 and NCQDs-3 electrodes were fabricated separately by mixing with conductive additive carbon black and a polymeric binder Poly (vinylidene fluoride) corresponding to 80:10:10 wt ratios. A gel-like paste was formed after the compositions were mixed with N-methylpyrrolidone (NMP) solvent in a mortar grinder. Afterwards, the active slurry formed was coated onto a graphite substrate. The dimensions of the graphite substrate were (2 × 1 cm) for the formation of the electrode in a three-electrode system, and a circular coin-shaped electrode was used for the two-electrode system. Finally, the electrodes were dried in the oven for 6 h at 80 °C to produce working electrodes. The active material was 0.0033–0.0024 g in a coin-shaped electrode and 0.002 g on a graphite strip (1 × 1 cm). The symmetrical supercapacitor devices were made by using NCQDs-1, NCQDs-2 and NCQDs-3 electrodes respectively, in a cell acting both as cathode and anode, separated by a micro-glass fiber in which a few drops of 1 M KOH were used as the electrolyte. The supercapacitor devices formed were named NCQDs-1, NCQDs-2 and NCQDs-3. However, for the symmetrical supercapacitor device NCQDs-1, NCQDs-2 and NCQDs-3, electrodes were used as cathodes and activated carbon electrodes were used as anodes, separated by a micro-glass fiber in which a few drops of 1 M KOH were used as the electrolyte.

### 2.5 Electrochemical measurements

Electrochemical studies of the devices were executed with the use of PGSTAT204 and PGSTAT30. The performances of the devices were investigated by cyclic voltammetry (CV) and Galvanostatic charge-discharge (GCD) between 0 and 0.6 V potential window using a scan rate range of 10 to 100 mVs^−1^ and at 0.1–0.5 A/g current densities. The capacitances ($\:{\text{C}}_{\text{S}}\:\text{a}\text{n}\text{d}\:\text{C}\text{p}$) (Fg^−1^) representing the supercapacitor electrodes were evaluated according to the equations:

1 $$\:{\:\text{C}}_{\text{S}}=\frac{\text{A}}{\text{m}\text{S}\varDelta\:\text{V}}$$

2 $$\:Cp=\frac{\text{I}\varDelta\:\text{t}}{\text{m}\varDelta\:\text{V}}$$

3 $$\:\:{\text{C}}_{\text{p}}=\:\frac{4\text{I}\varDelta\:\text{t}}{\text{m}\varDelta\:\text{V}}$$

Where A denotes the area beneath the CV curves, $\:\text{m}\:$denote active mass (g), S denotes the scan rate (mV/s), $\:\varDelta\:\text{V}$ represents the potential range (V), I (A) depicts the discharge current and $\:\varDelta\:\text{t}$ (s) represents discharge time. Equation (1) is used to calculate the specific capacitance from the CV curve and Eqs. (2) and (3) is used to calculate the specific capacitance from the GCD curve in the three-electrode system and the two-electrode system.

## 3. Results and discussion

**Fig. 2:**
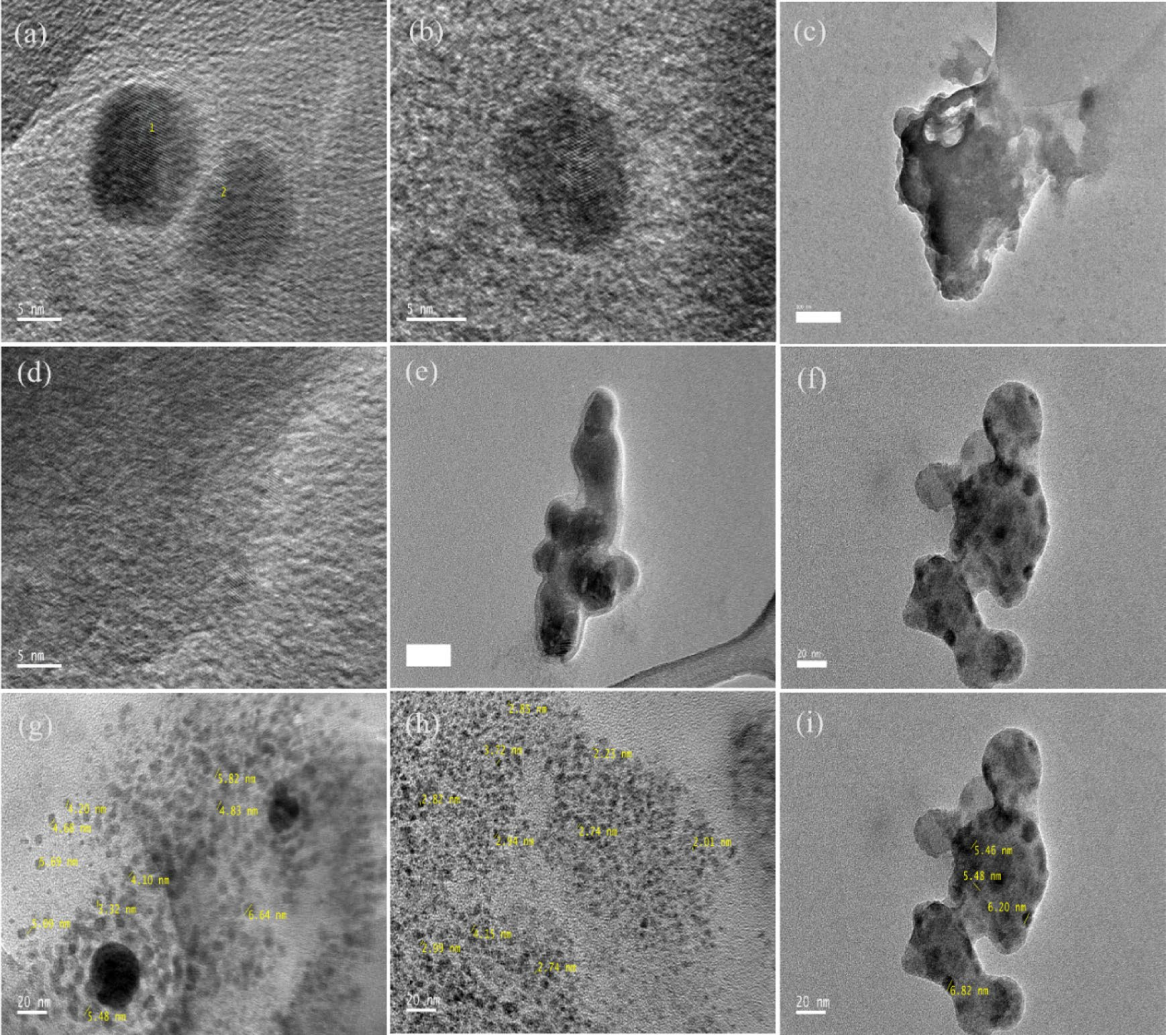
HR-TEM images (**a**, **b**) NCQDs-1 (**c**, **d**) NCQDs-2 (**e**, **f**) NCQDs-3 and (**g**-**i**) fringe width of the NCQDs-1, NCQDs-2 and NCQDs-3.

**Fig. 3:**
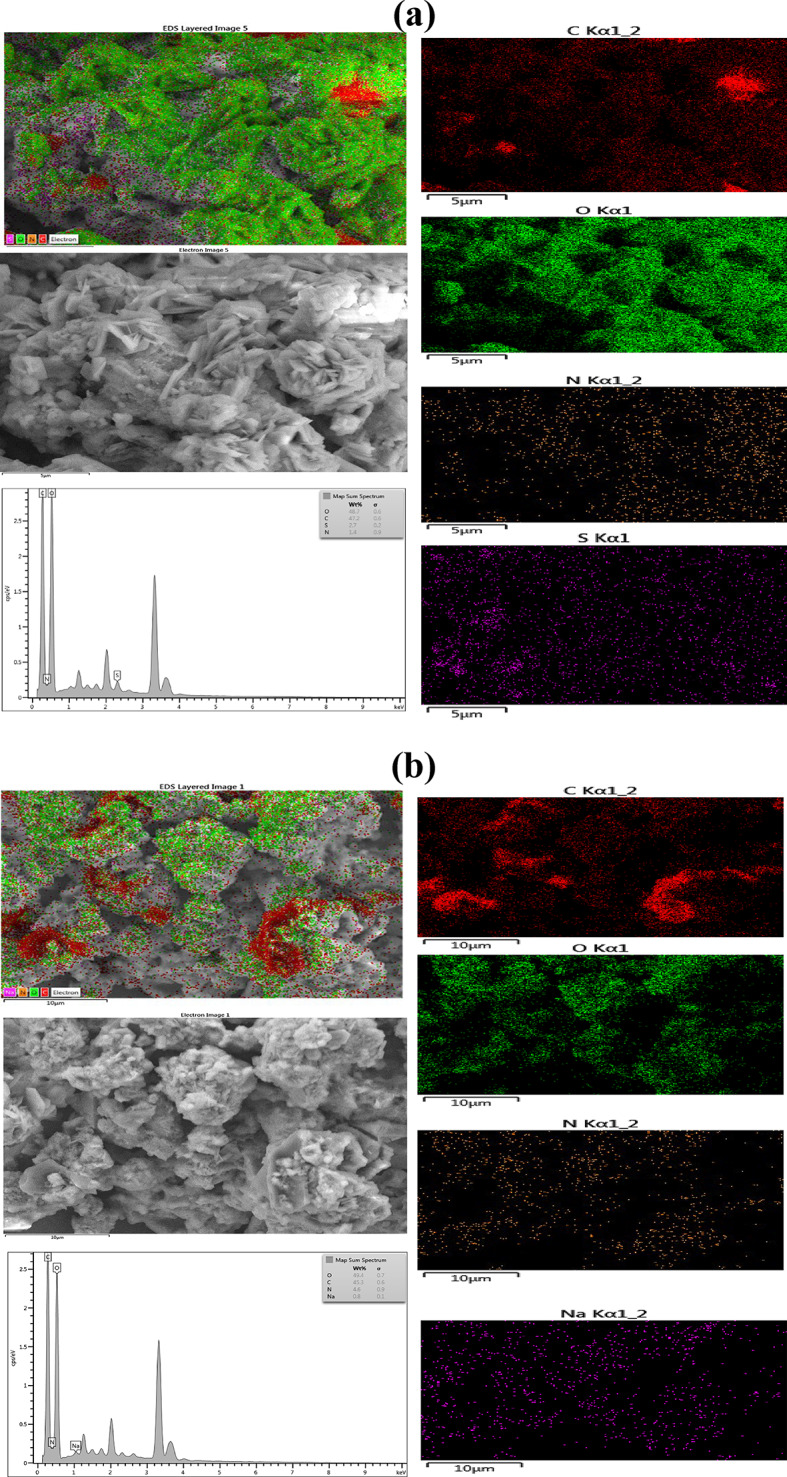

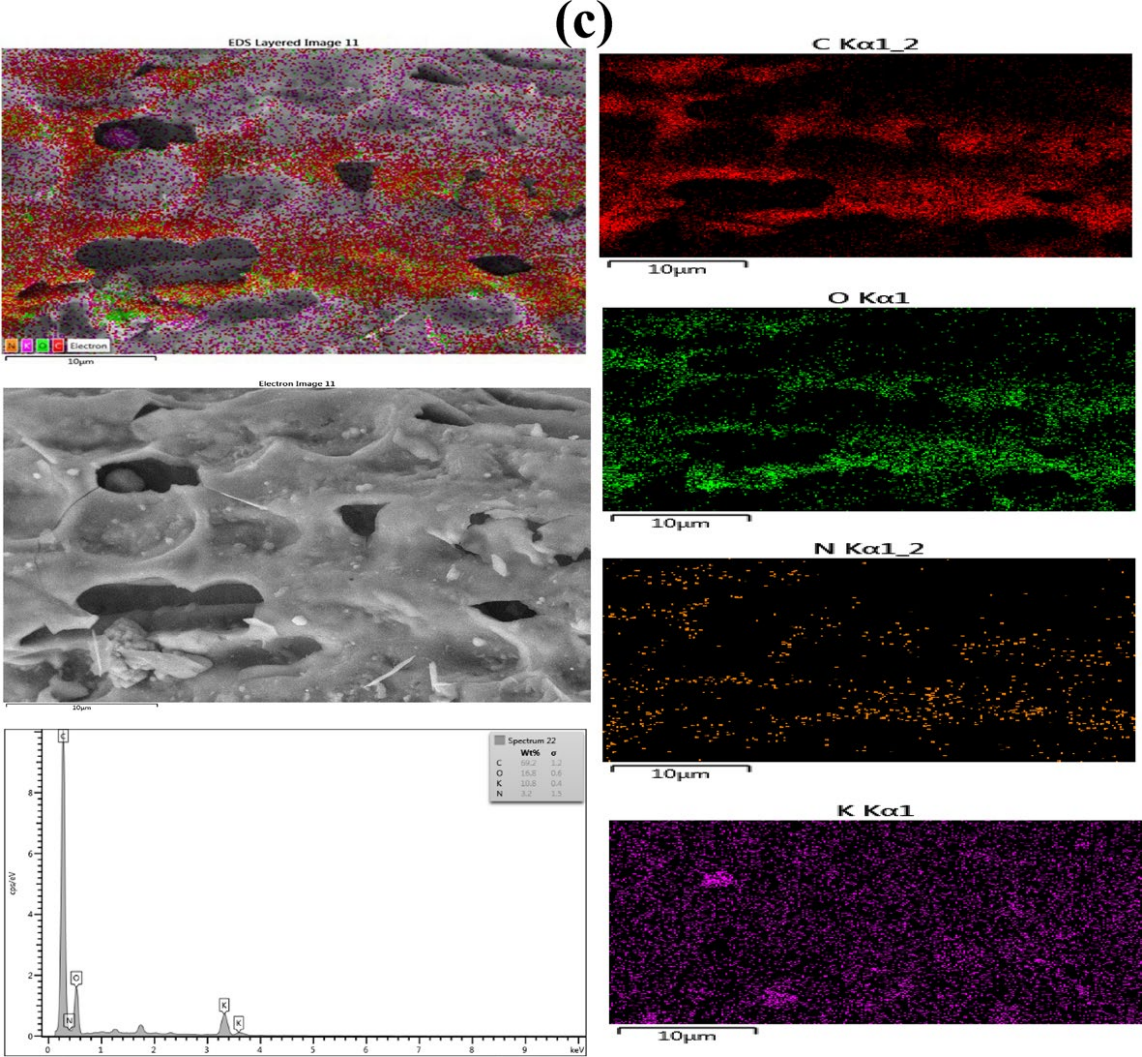
(**a**) Elemental mapping with EDX spectra of the NCQDs-1. (**b**) Elemental mapping with EDX spectra of the NCQDs-2. (**c**) Elemental mapping with EDX spectra of the NCQDs-3.

The morphology of the active materials was examined using HRTEM. Figure 2 shows the HRTEM images, which denote monodispersed spherical-shaped particles (Fig. 2a-f). The HRTEM images indicated from the particle size distribution a demonstration of d-spacing of the electrodes as 0.215, 0.28, and 0.271 nm, respectively (Fig. 2g-i). From the low and high magnification (20 and 5 nm) of the HRTEM images, respectively, the NCQDs-1, NCQDs-2 and NCQDs-3 particles exhibited uniform dispersion without obvious lattice fringes, which is consistent with the previous reports, comparatively based on the morphology and the ranges of the nanoparticle sizes^1–3^. The monodispersed HRTEM images show spherical geometry with a particle size of 2 to 7 nm. Figure 3(a-c) depicts the elemental mapping with EDX spectra of the NCQDs-1, NCQDs-2 and NCQDs-3. The compositions revealed that the major elements were carbon, oxygen, and nitrogen. The surface morphology reflected the influence of the activating agents on the precursory carbon-sourced materials, and the effect of the N-doping of the active materials can be attributed to the pores and deformities comprising interlayer spacings, which can enhance pore diffusion of electrolytes and can create an area for additional ion storage. This work proves successful nitrogen doping, which is a basic process in the surface characteristic modification of the material. Nitrogen doping and activating agents influence the surface structure by the formation of pores and structural defects, which increase the interlayer spacings. These properties are important since they facilitate electrolyte diffusion across the pores and increase the surface area accessible for the ion storage mechanism, which is necessary to improve the performance of electrochemical devices such as batteries and supercapacitors.

**Fig. 4:**
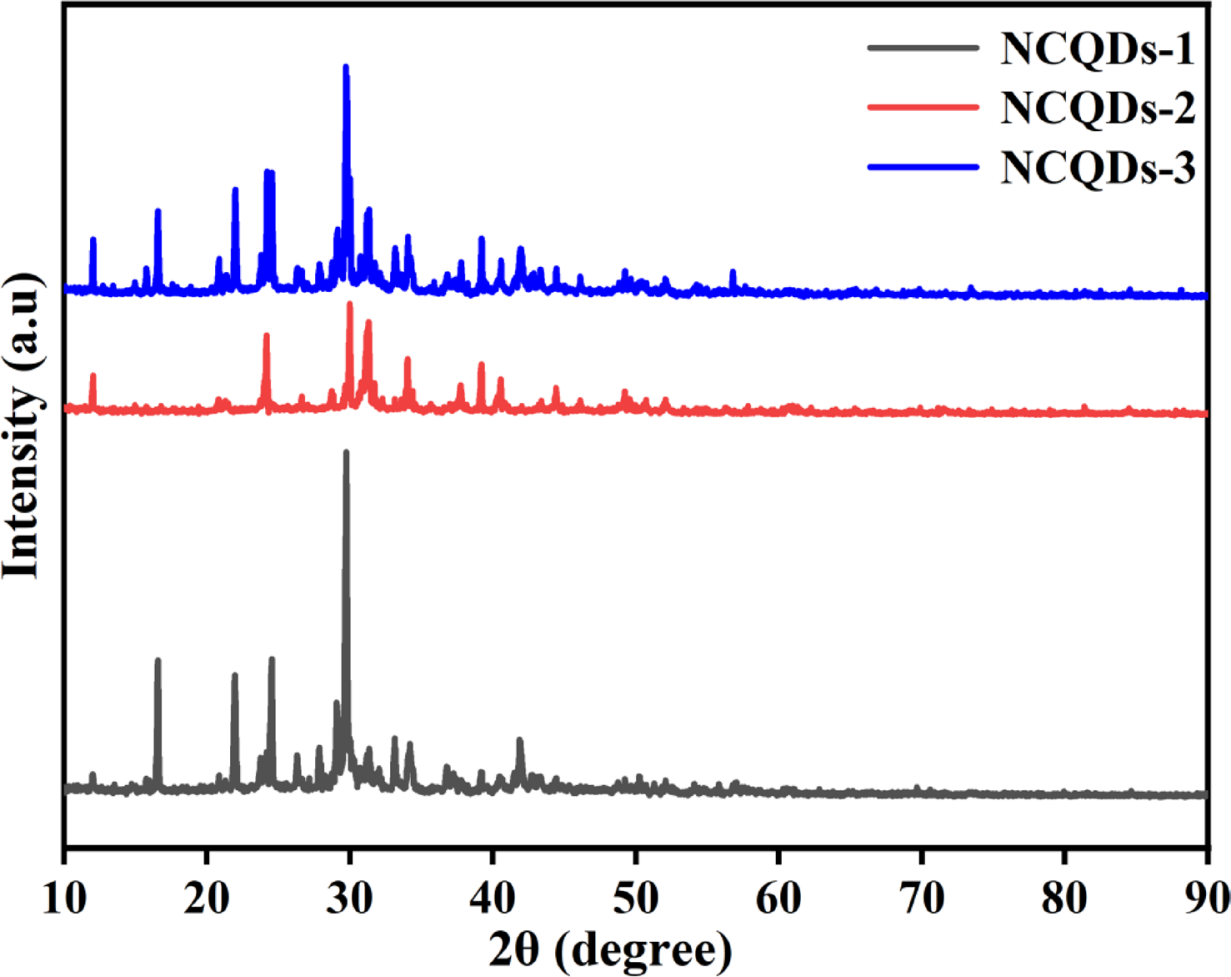
XRD spectra of the NCQDs-1, NCQDs-2, and NCQDs-3.

The crystallinity of the NCQDs-1, NCQDs-2 and NCQDs-3 from different activation conditions is confirmed in Fig. 4. It can be inferred that typical diffraction for all samples appeared around 25^o^ to 44^o^ corresponding to (hkl) crystallographic planes of (101) and (112) for NCQDs-1, (004) and (117) for NCQDs-2 and (110) and (117) for NCQDs-3^29^. This indicates the amorphous structure in the carbon material exhibited crystal reflection of graphitic framing, which was influenced by the activation process of the precursory material, with average inter-spacings of 3.95 nm, thereby reinforcing the conclusions obtained from the HRTEM studies^56,65^. Among these, prominent peaks for all samples at 24.1º and 30.03º denoted SP^2^ graphitic structure, while peaks at 38.9^o^ were indicative of SP^3^ diamond structure^66^. The NCQDs-1, NCQDs-2 and NCQDs-3 presented a remarkable diffraction peak, which was noticed around 25^o^ for the materials attributed to their crystallinity structures. The peak at 27.1° denoted the formation of a graphitic network of NCQD materials, indicative of interlayer spacing via carbonization of CAS^67^. The weak reflection peaks were noticed for all samples when 2θ = 40–50º, indicating crystalline disorder attributed to the defects due to the nitrogen and oxygen functional groups in the basal plane, which were formed during the processing of the NCQDs^68^. The peak at 29.5º is observed for NCQDs-1, NCQDs-2 and NCQDs-3. The intensity of this peak is very high in NCQDs-3 compared to NCQDs-2 and NCQDs-1. This indicates an increased graphitization degree and the random lattice strain of the carbon matrix during activation. This determines that the carbon atoms have been arranged into more graphitic (sp²-bonded) structures, which improves the material’s electrical conductivity and stability. Additionally, the high intensity of the peak is linked to random lattice strain in the carbon matrix, which occurs during the activation process. The activation introduces defects, pores, and distortions in the carbon framework, contributing to strain and influencing the structural arrangement.

**Fig. 5:**
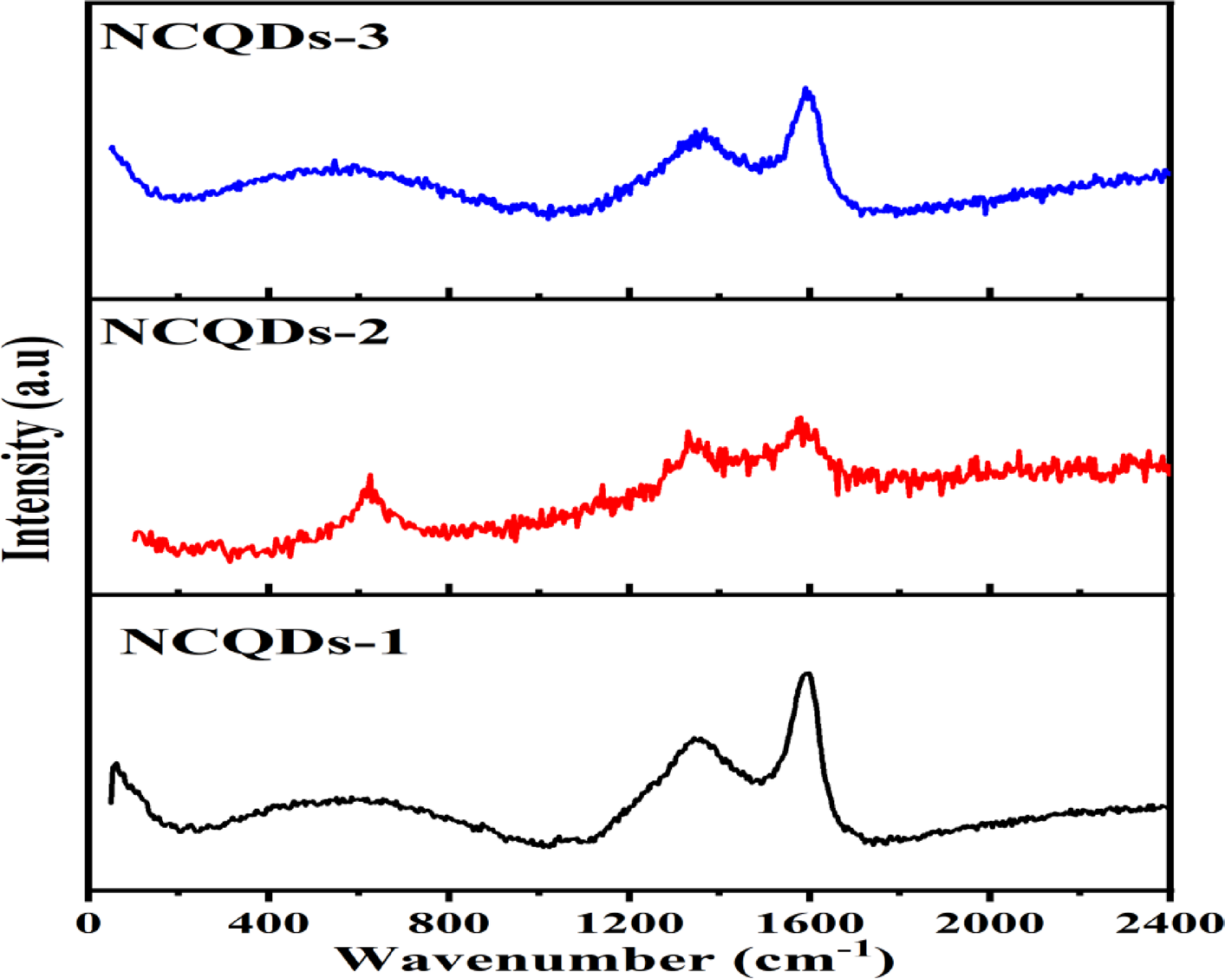
Raman spectra of NCQDs-1, NCQDs-2, and NCQDs-3.

The chemical structure and composition of the materials are depicted using Raman spectra (Fig. 5). The NCQDs-1 revealed two broad peaks at 1347.08 and 1593.31, denoting D bands (SP^3^) and G bands (SP^2^). Similar spectra were obtained for NCQDs-2 and NCQDs-3, with D bands 1337.31 and 1357.47.6. The G bands were 1603.69 and 1583.75, respectively. In general, the Raman spectra data indicated that SP^3^ and SP^2^ hybridized carbon defects were predominant in the NCQD materials^69^. The intensity ratio notably confirmed that the disordered D and G (ID/IG) bands were 0.845, 0.834 and 0.857, belonging to the carbon^70,71^. This demonstrates that CQDs possess minimal surface defects after the N-doping^72^. Thus, N doping is successfully done in the NCQDs-1, NCQDs-2, and NCQDs-3.

**Fig. 6:**
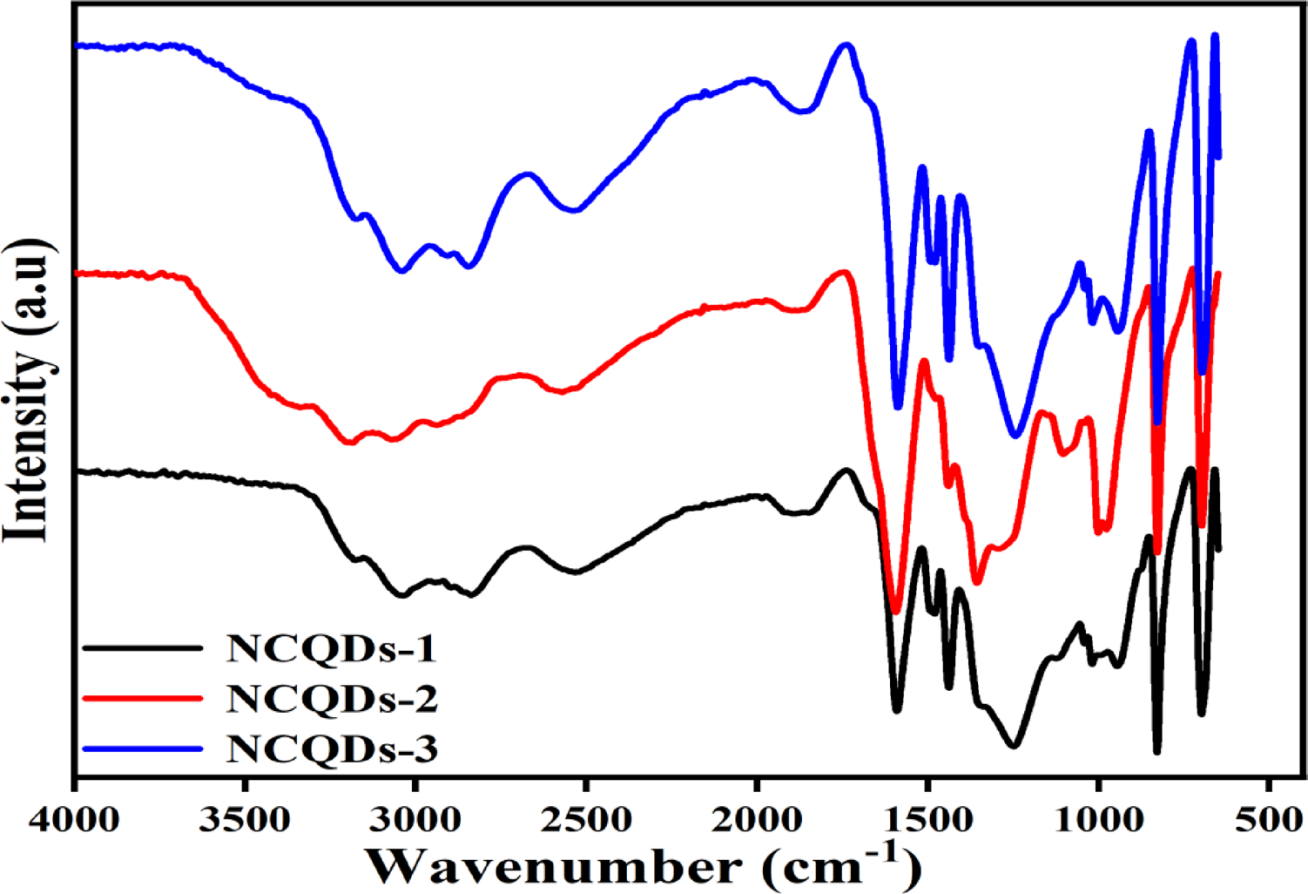
FT-IR spectra of the NCQDs-1, NCQDs-2 and NCQDs-3.

The FT-IR spectroscopic analysis depicts the significant surface functional groups on the prepared NCQDs-1, NCQDs-2 and NCQDs-3 (Fig. 6). The adsorption bands around 3185, 3307, and 3171 (cm ^−1^) were assigned to the hydroxyl groups^73^. The central bands around 3054, 2994, 3068 and 3041, 2910 (cm^−1^) were assigned to the O-H vibrations^19,20^. Notably, in all functional properties of the NCQDs materials, spectra bands between 2944 and 2910 (cm^−1^) were ascribed to the SP^3^ C–H vibrations. The NCQDs exhibited C-N stretching vibrations at 1450 cm^−1^, indicating the doping of N elements on the interface of CQDs^74,75^. The band at 1400 cm^−1^ for all samples was assigned to the O-CH and C-OH groups, which reflected that the increase in carbonization temperature and activation time of CAS inhibited surface agglomeration^76^. The peak around 1620 cm^−1^ for all samples was assigned to C = C stretching vibration, denoting SP^2^-hybridized carbon, which proved the availability of the carbonyl group^77^. The peaks at 1520 cm^−1^ − 1265 cm^−1^ assigned to N-H and C-N indicated amine groups on NCQDs due to the O- and N-groups^78^. It also proved that the doping of nitrogen to achieve NCQDs-1, NCQDs-2 and NCQDs-3 structures contained numerous active functional groups denoted by N-C = O and C-N-C at bands of 1610, 1510, 1412, 1330 (cm^−1^)^79,80^. Furthermore, the surface of these structures indicated sharp peaks at 675 cm^−1,^ indicating the presence of -NH_2_, thereby reinforcing that the doping of Nitrogen on the core shells of the CQDs was successful^81^.

**Fig. 7:**
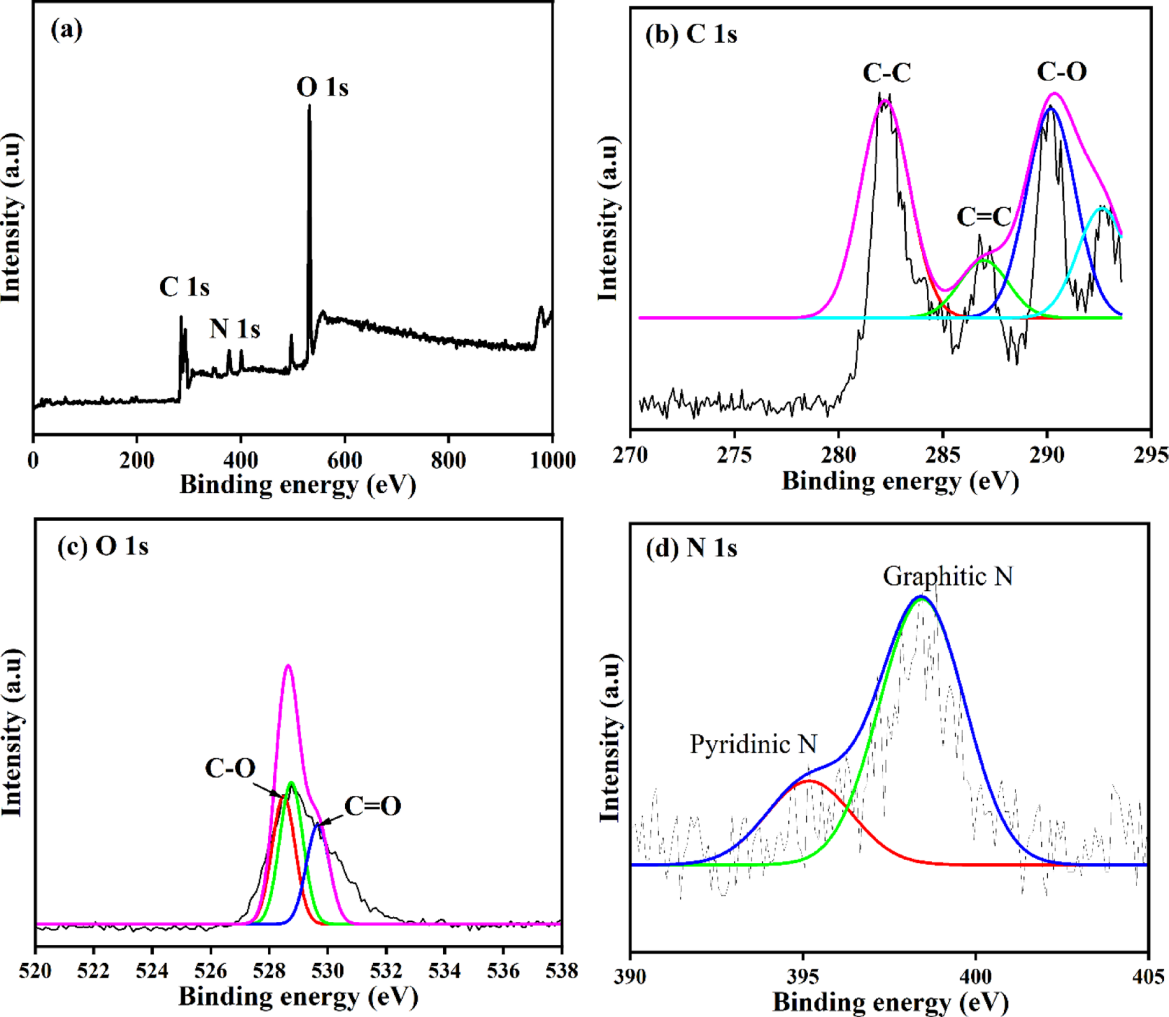
**(a)** XPS survey spectra **(b)** C-1s, **(c)** O-1s, and **(d)** N-1s of the NCQDs-3.

Further, to find out the exact elements present in the NCQDs-1, NCQDs-2 and NCQDs-3, XPS studies of NCQDs-3 were done. Figure 7(a) shows the full XPS spectra of the NCQDs-3. The full XPS spectra of the NCQDs-3 show three dominant peaks denoting O-1s, C-1s, and N-1s elements. The peaks are seen at 285.04, 400.07, and 533 eV, respectively, indicating successful N-doping on the CAS-derived CQDs. Figure 7(b) shows the deconvoluted curves of the C1s peaks indicating the availability of C-C, C = C, and C = O^42^. The high-resolution O-1s spectra were obtained between 524 and 536 eV, corresponding to the presence of C = O and C - O groups **(**Fig. 7c**)**. Meanwhile, the spectra of N-1s spectra were as a result of C-N bonding and amine groups denoting the graphitic and pyridinic surface **(**Fig. 7d). In general, the spectra depict that the obtained CQDs comprise structures of integrating particle sizes primarily denoting SP^2^ carbon and N-doped urea source^82^.

## 4. Electrochemical studies

**Fig. 8:**
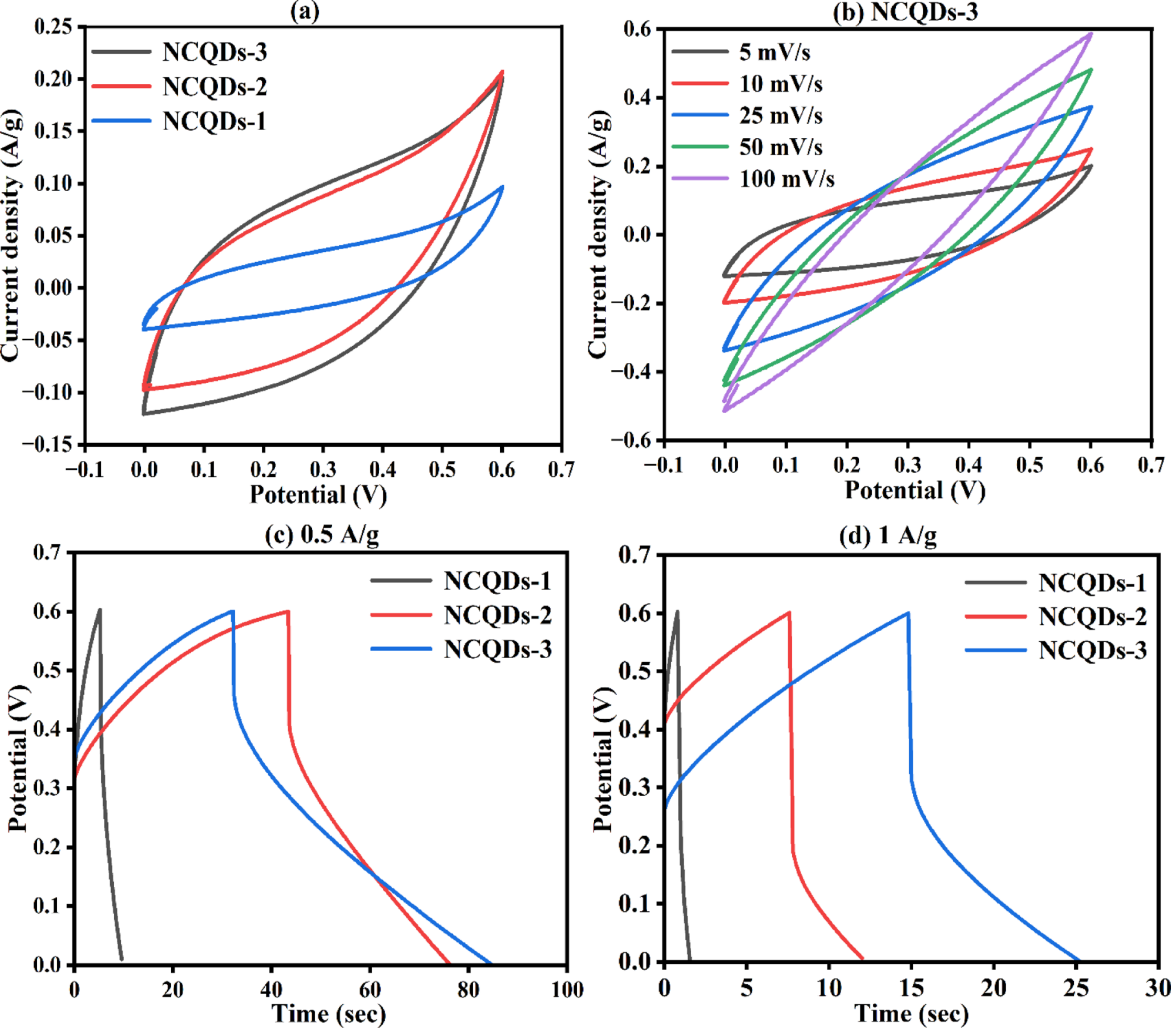
**(a)** CV curves of the NCQDs-1, NCQDs-2 and NCQDs-3 electrodes at the scan rate of the 5 mV/s **(b)** CV curves of the NCQDs-3 electrodes at the scan rate of 5-100 mV/s **(c**,** d)** GCDs curves of the NCQDs-1, NCQDs-2 and NCQDs-3 electrodes at the current density of 0.5 and 1 (A/g).

**Fig. 9:**
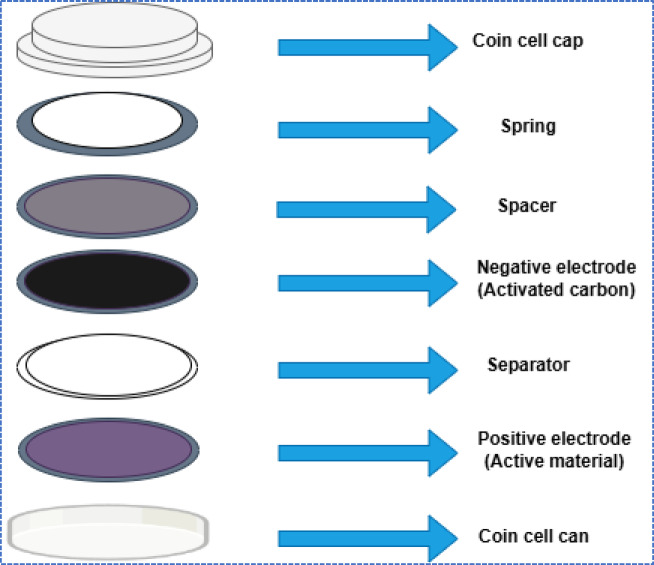
Schematic diagram of asymmetric coin cell.

The electrochemical performance of the fabricated NCQDs-1, NCQDs-2, and NCQDs-3 electrodes was checked in a three-electrode system and in a two-electrode system. The electrochemical performance of electrodes was studied through CV, GCD, EIS, and cyclic stability tests. Figure 8 (a) shows the CV curves of the NCQDs-1, NCQDs-2, and NCQDs-3 electrodes in a three-electrode system at a scan rate of 5 mV/s. The CV curves do not show any reduction peaks, demonstrating the EDLC nature of the NCQDs-1, NCQDs-2 and NCQDs-3 electrodes. In EDLCs, the charge storage mechanism is primarily governed by electrostatic adsorption of electrolyte ions at the electrode/electrolyte interface. The high surface area of NCQDs-1, NCQDs-2 and NCQDs-3 electrodes facilitates a large number of accessible active sites, enhancing the double-layer capacitance. When potential is applied, ions from the electrolyte are physically adsorbed onto the surface of the NCQD material electrodes, forming a Helmholtz double layer. This process is highly reversible and does not involve electron transfer or chemical transformation, ensuring excellent cycling stability. The presence of nitrogen functional groups in NCQDs may contribute to increased wettability and improved conductivity, further supporting efficient ion transport and EDLC behaviour. Figure 8 (b) shows the CV curves of the NCQDs-3 electrodes at the scan rate of 5-100 mV/s and Fig. 8 (c, d) shows the GCDs curves of the NCQDs-1, NCQDs-2 and NCQDs-3 electrodes at the current density of 0.5 and 1 (A/g). The specific capacitance values calculated using Eq. (1) are 4, 25 and 43 (F/g) through the CV curve and using Eq. (2) are 3, 8 and 16 (F/g) through the GCD curves at the current density of 0.5 A/g of the NCQDs-1, NCQDs-2 and NCQDs-3 electrodes. The NCQDs-3 exhibited enhanced electrochemical performance in KOH electrolyte due to their high ionic conductivity and smaller hydrated ion size (K⁺), which facilitates rapid ion diffusion and efficient double-layer formation. This leads to improved charge transport kinetics and higher specific capacitance, as reflected in both CV and GCD analyses. See Fig. 9.

### 4.1 Asymmetrical coin cell

**Fig. 10:**
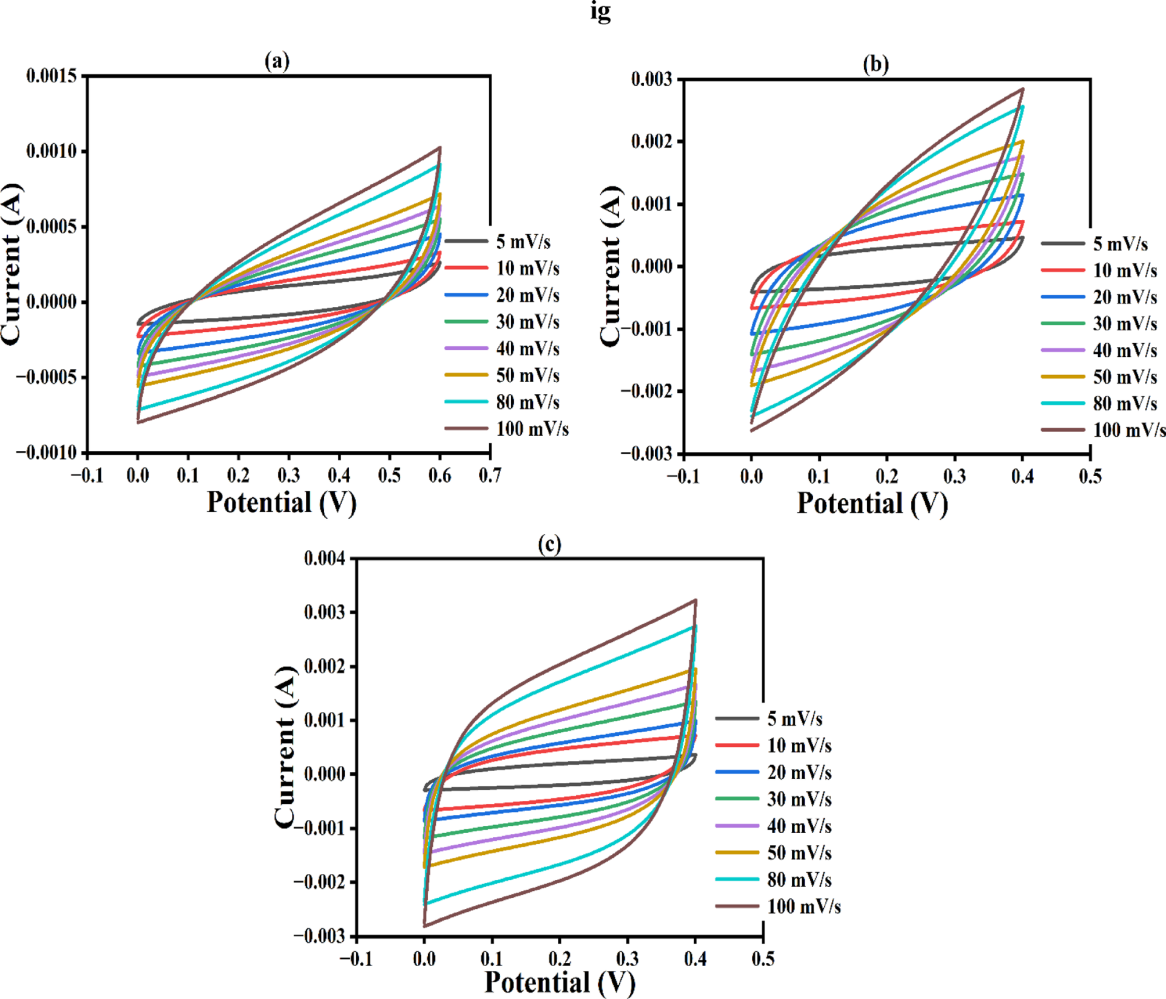
**(a-c)** CV curves of the NCQDs-1, NCQDs-2, and NCQDs-3 electrodes.

The potential of the fabricated electrodes for practical application as a supercapacitor device was determined by assembling and testing the asymmetric supercapacitor (ASC) using the NCQDs-1, NCQDs-2 and NCQDs-3 electrodes as positive electrodes (cathode) while a commercial carbon served as the negative electrode shown in Fig. 8. To evaluate the performance of the electrodes, the CV and GCD cyclic studies were examined. The CV curves were obtained at a scan rate of 5–100 mVs^–1^ and were suggestive that the assembled ASC could function by utilizing the voltage range between 0 and 0.6 V. Figure 10 (a-c) denotes the CV studies of the NCQDs-1, NCQDs-2 and NCQDs-3 electrodes. As depicted by the CV curves of the devices, some distortions from the ideal rectangular shapes were noticed. The deformed shapes in nature were formed as a result of the EDLC of the negative electrode^83,84^. It can be noticed that the ASC exhibited stability up to 0.6 V potential window as denoted by quasi CV rectangular shapes, which were noticeably retained, indicative of good supercapacitive behaviour^85^.

**Fig. 11:**
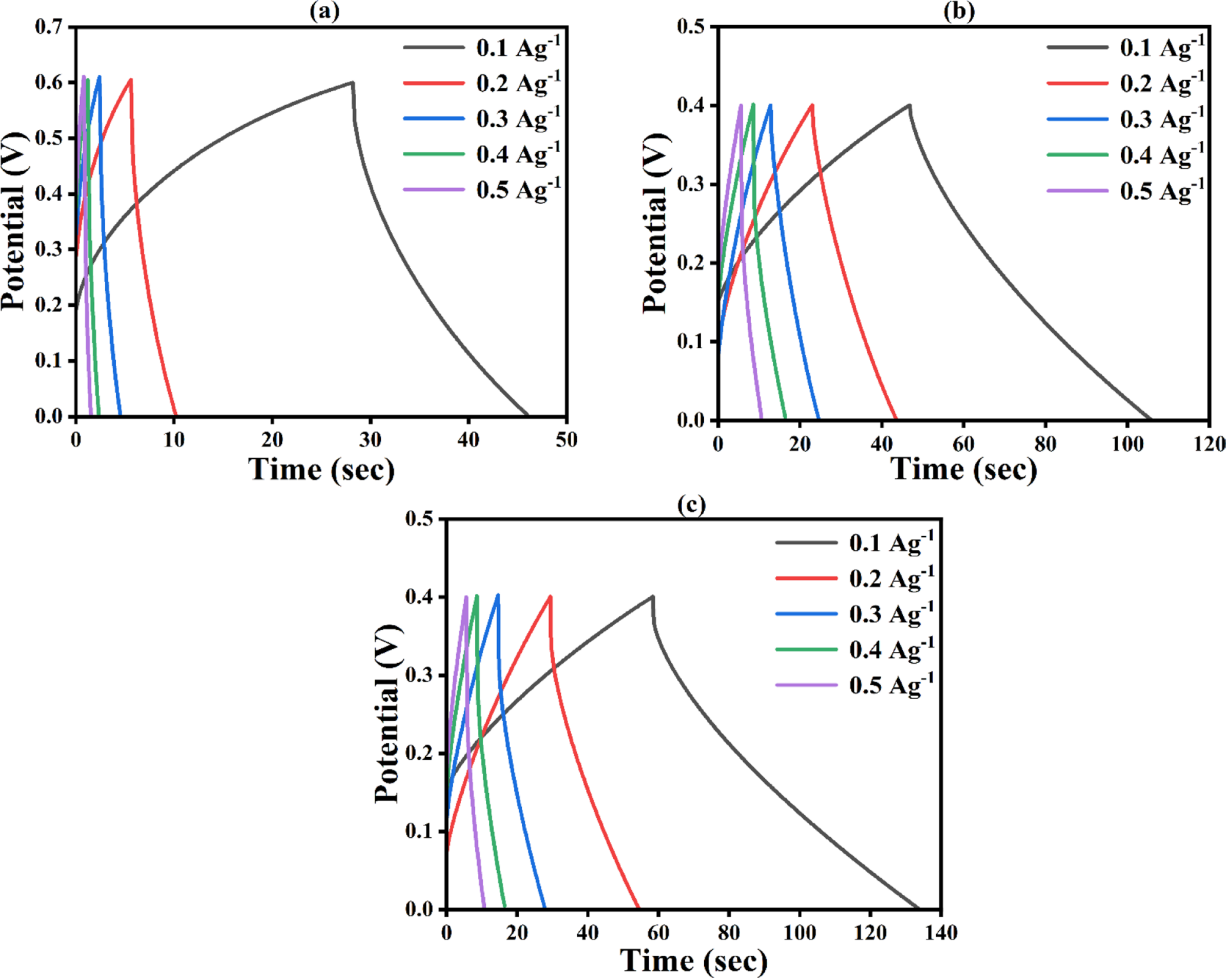
**(a-c)** GCD curves of the NCQDs-1, NCQDs-2, and NCQDs-3 electrodes.

Figure 11 (a-c) shows the GCD curves of the NCQDs-1, NCQDs-2, and NCQDs-3 electrodes. The GCD profiles indicate that NCQDs-1, NCQDs-2 and NCQDs-3 electrodes, as a function of voltage over time, exhibit ideal semi-triangular shapes of electrodes. The specific capacitance values were evaluated at 0.1 to 1 Ag^−1^ current densities. It observes that as the current density increases, there is a noticeable increase in the IR drop, which refers to the internal resistance effect that reduces the effective voltage during discharge. However, at lower current densities (0.1 A/g), the voltage drops more gradually, indicating that the electrodes are better at retaining their capacitance, meaning they can store and release more charge efficiently under these conditions. Among the three electrodes, NCQDs-3 showed the best performance, achieving a specific capacitance of 15 F/g, while NCQDs-1 and NCQDs-2 reached 12 F/g and 4 F/g, respectively. This suggests that the materials used to prepare these electrodes, particularly the doping with nitrogen, played a crucial role in improving their conductivity and overall performance, even when using a lower concentration of KOH electrolyte. Additionally, the superior electrochemical behavior of NCQDs-3 is attributed to its well-developed and tunable pore network, which enhances the movement and diffusion of ions in the electrolyte, ultimately boosting the electrode’s energy storage capabilities.

### 4.2 Symmetrical cion cell

**Fig. 12:**
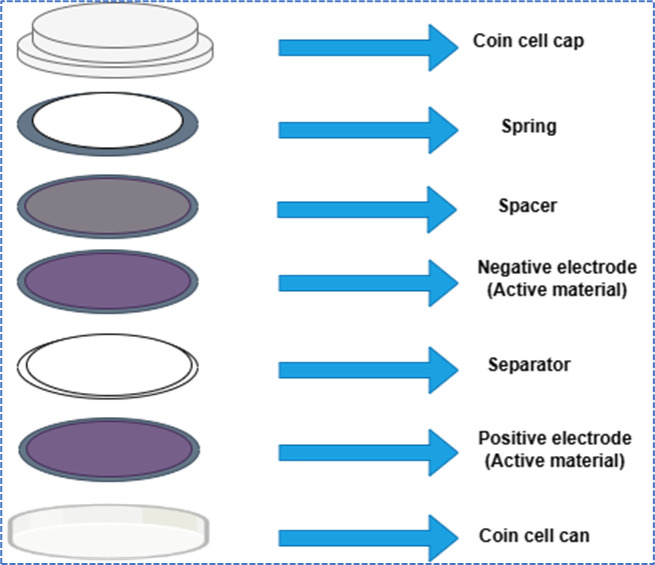
Schematic diagram of the symmetrical coin cell.

The electrochemical performance of symmetric cell (SC) devices produced from NCQDs-1, NCQDs-2, and NCQDs-3 electrodes is explained by using CV, GCD, EIS, and cyclic stability tests. The schematic diagram of a symmetrical coin cell is shown in Fig. 12.

**Fig. 13:**
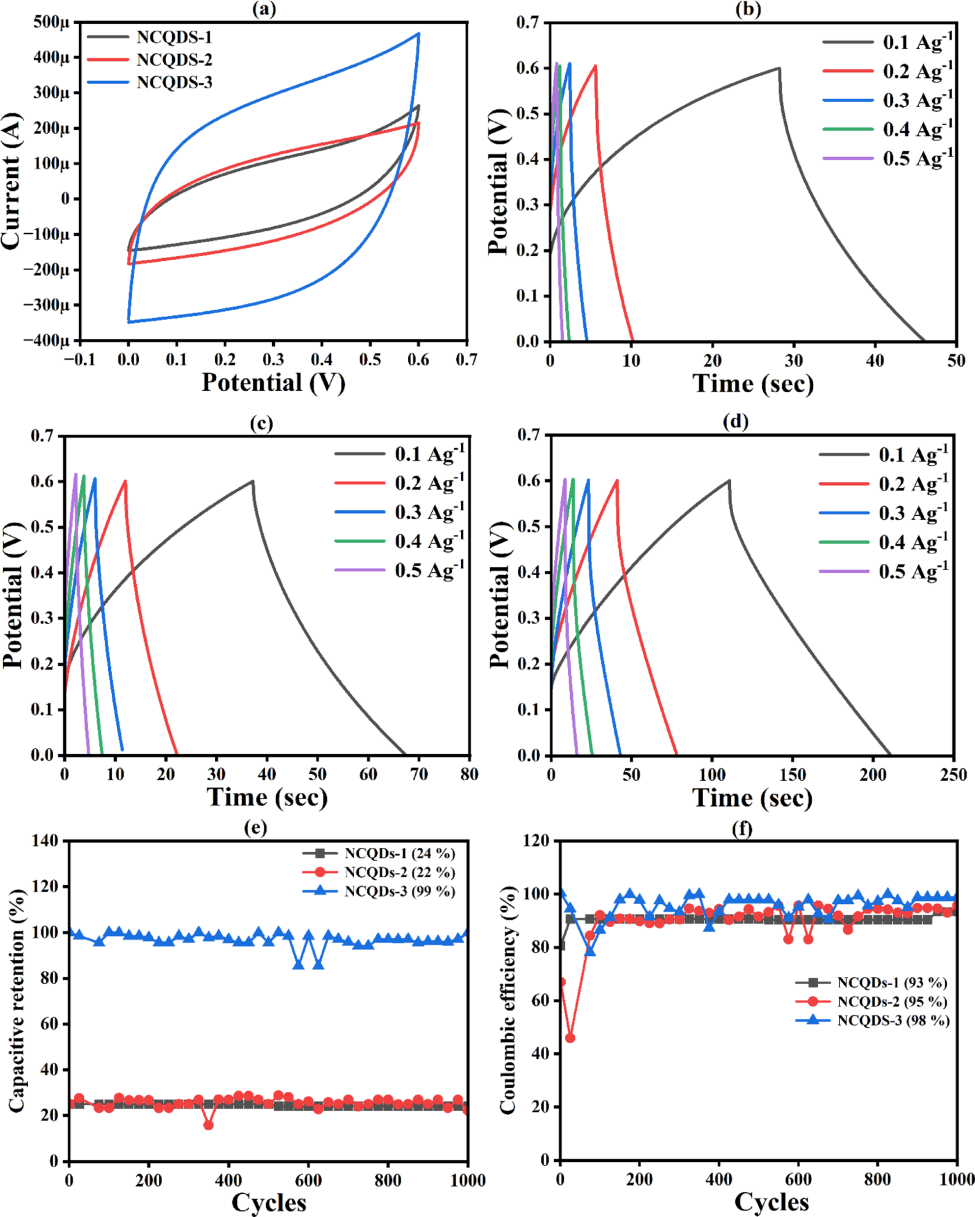
**(a)** CV curves **(b-d)** GCD curves **(e)** capacitive retention and **(f)** coulombic efficiency of the NCQDs-1, NCQDs-2, and NCQDs-3 electrodes. Schematic diagram of the symmetrical coin cell.

Figure 13 (a) denotes the CV curves of the NCQDs-1, NCQDs-2, and NCQDs-3 electrodes at a scan rate of 5 mV/s. The CV curves of the NCQDs-1, NCQDs-2, and NCQDs-3 electrodes were obtained within the potential range of 0 to 0.6 V. Within the experimental scan rates, the CV curves maintained similar shapes, with the highest CV curves obtained by the NCQDs-3 electrode. Figure 13 (b-d) shows the GCD curves of the NCQDs-1, NCQDs-2, and NCQDs-3 electrodes at the current densities of 0.1, 0.2, 0.3, 0.4 and 0.5 (A/g). The specific capacitance of each of the NCQDs-1, NCQDs-2, and NCQDs-3 electrodes was determined from the area covered by the GCD curves as a function of the discharge time according to Eq. (2). At a specific current 0.1 Ag^−1^, the highest specific capacitance of 22 Fg^−1^ was obtained from NCQDs-3, which was comparatively higher than a commercial supercapacitor formed from a symmetric activated carbon device. The electrochemical characteristics of a device produced from commercial activated carbon under full cell conditions were found to be within 17 F/g to 20 F/g^16,86^. The cell could still retain its original value within the specific current of 0.2–0.5 Ag^−1^. This can be attributed to the positive effect of adding NCQD materials on the improvement of the capacitive behavior of the cells. It can be seen that the GCD curves were triangular, indicating consistency with the EDLC features of a symmetric device. The GCD plots could still retain a symmetrical triangular shape at 0.5 Ag^−1^ current density, demonstrating that the devices possess efficient reversible energy storage properties^87^. From the GCD curves, the capacitance for the electrodes indicates that the incorporation of NCQDs in the cell structure potentially increased the capacitance of the SC electrodes. Figure 13e shows the capacitive retention and Fig. 13(f) shows the coulombic efficiency of the NCQDs-1, NCQDs-2, and NCQDs-3 electrodes. The NCQDs-1, NCQDs-2, and NCQDs-3 electrodes attain the coulombic efficiency of 93, 95 and 98 (%) and a capacitive retention of 24, 22 and 99 (%) even after 1000 GCD cycles. The improved electrochemical performance of the NCQDs-3 electrode compared to NCQDs-2 and NCQDs-1 can be attributed to several significant factors related to the synthesis environment. Being a strong base, KOH allows for improved surface passivation as well as the development of rich surface functional groups from the doping source (urea), particularly oxygen and nitrogen functional groups, responsible for electron transfer during charge-discharge processes. This leads to enhanced coulombic efficiency and capacitive retention. The KOH etching also creates a more porous carbon structure with a higher specific surface area, enabling better penetration of the electrolyte and ion diffusion, ultimately leading to improved available electroactive sites for charging storage. Therefore, NCQDs-3 show an optimal balance of surface chemistry, porosity, and stability, which leads to considerably improved coulombic efficiency (98%) and capacitive retention (99%) after 1000 GCD cycles relative to NCQDs-2 and NCQDs-1 electrodes. The specific capacitance of materials, which include activated carbon, carbon nanotubes (CNT), exfoliated graphite, and CQDs in a two-way electrode system, is compared with the present studies is shown in Table 1.

**Fig. 14:**
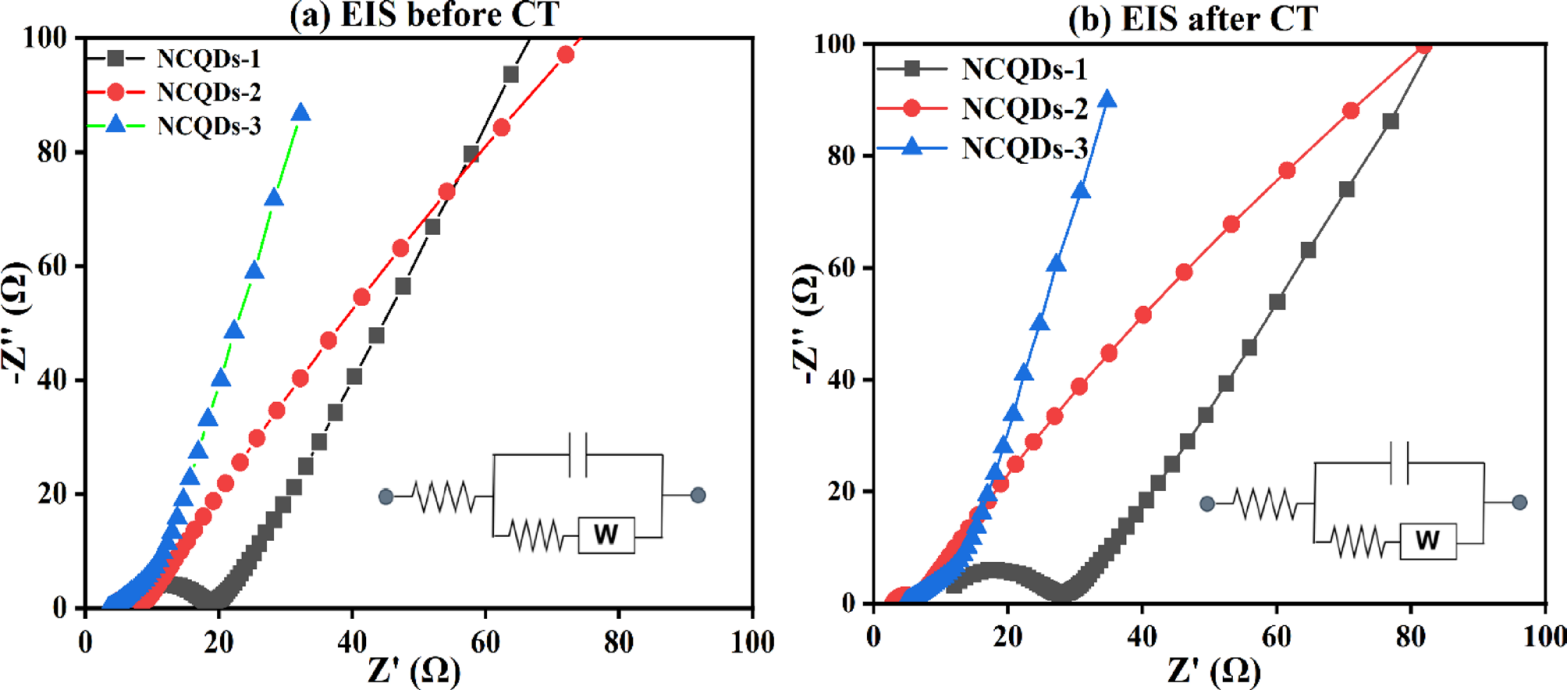
(**a**, **b**) Nyquist plot before and after cyclic stability test of the NCQDs-1, NCQDs-2 and NCQDs-3 electrodes.

EIS is a powerful technique used to understand the internal resistances and charge storage mechanisms within electrochemical systems. Figure 14(a) and (b) present the Nyquist plot of the NCQDs-1, NSQDs-2 and NCQDs-3 electrodes before and after cyclic stability tests. The Nyquist plot of NCQDs-1, NCQDs-2 and NCQDs-3 electrodes graph shows the real part of impedance versus the imaginary part over a frequency range of 10 mHz to 100 kHz. In these plots, the semicircular arc observed at high frequencies is typically associated with the charge transfer resistance (R_ct_) at the electrode-electrolyte interface, the linear straight line at low frequencies is the capacitive behavior, which corresponds to ion diffusion in the electrode pores (usually called Warburg impedance). The results of the Rs values are 6.27, 4.10 and 3.35 (ohm) and Rct are 12, 4.56 and 0.96 (ohm) for the NCQDs-1, NCQDs-2 and NCQDs-3 electrodes in a symmetrical cell pattern before cyclic stability tests. The increase in Rs and Rct values after the cyclic stability test can be attributed to structural degradation and electrode/electrolyte interface changes. The prolonged cycling may lead to surface passivation, electrode material dissolution, or morphological changes such as cracking or loss of active surface area, which hinder ion transport and electron transfer. Additionally, the accumulation of electrolyte decomposition products or the formation of resistive layers at the electrode surface can elevate interfacial resistance. These factors collectively contribute to the observed increase in Rs and Rct values after the cyclic stability tests of the NCQDs-1, NCQDs-2 and NCQDs-3 electrodes.

**Table 1:** Comparison of specific capacitance values with the literature report.

Material	Scan Rate (mV·s − 1)	Tested Potential Window (V)	Electrolyte	Capacitance (F/g)	Reference
Activated carbon aerogel	-	0.8–1.6	1 M NaCl	19.7	^88^
CNT	5–50	0–1	1M H_2_SO_4_	28	^89^
Commercial Activated Carbon	5 to 200	1.9	0.5 H2SO4	15	^90^
Commercial Activated Carbon	30	0.1	1 M (C2H5)4NBF4/propylene carbonate	17	^16^
Activated carbon nanofiber	-	0–1	6 M KOH	18	^91^
Exfoliated graphite	3	0–1	0.5 M NaPF6	4.22	^92^
Graphitic carbon nitride	-	−1–1	Na2SO4	11	^93^
Poly (ethylene glycol) monomethyl ether acrylate	10	0–3	ionic liquids	9.5	^94^
Composite solid polymer electrolytes	10	0–3	ionic liquids	10	^94^
Nitrogen doped CQDs/Cu	-	0.05–3	1:1 ethylene carbonate and dimethyl carbonate	11.5	^48^
Callerya Atropurpurea Shells NCQDs	5-100	0-0.6	1 M KOH	22	This study

### Research findings

This particular study effectively illustrated the successful synthesis of nitrogen-doped carbon quantum dots (NCQDs) through the shells of Callerya Atropurpurea via a hydrothermal method. This process involved the chemical activation of NCQDs using various agents, i.e., KOH, H₂SO₄, and NaOH. Out of these activation agents that were tested, KOH proved to be the most suitable one, which ended up significantly enhancing the electrochemical performance of the NCQD electrodes.

The findings from the experiments disclosed an important result: the KOH-activated nitrogen-doped carbon quantum dots, which are designated as NCQDs-3, exhibited the highest performance upon assessment based on both specific capacitance and cycling stability among all the materials that were tested. Particularly, it is notable to mention here that the NCQDs-3 electrode showed an excellent coulombic efficiency rate of 98% in addition to a very good capacitive retention of 99% after experiencing 1000 charge and discharge cycles. This superior performance significantly points towards excellent stability in the long term, along with a very good energy storage capacity.

The significantly enhanced performance of the NCQDs-3 can be mainly attributed to the formation of a highly complex and porous structure with numerous available active sites. This unique structural characteristic effectively facilitated quicker ion diffusion processes and provided a more effective charge transfer process. Besides, the addition of nitrogen doping in the system brought additional capacitive behaviour, which also played a beneficial role in delivering an enhanced electrochemical response along with greater extents of conductivity. In comparison with the NCQDs activated using H₂SO₄ and NaOH, the samples activated using KOH were found to possess much more desirable structural properties along with better surface properties. These positive attributes cumulatively make the KOH-activated samples far more suitable for utilization in supercapacitor applications. These results not only shed light on the promise of Callerya Atropurpurea shells as a low-cost, renewable source of carbon but also underscore the fundamental importance of activation agents in controlling the structure and functional properties of NCQDs. The outstanding performance of the KOH-activated NCQDs points toward a viable pathway for the synthesis of high-performance, biomass-derived electrode materials for energy storage devices.

## Conclusion

In this study, CQDs were synthesized through the hydrothermal technique using *Callerya Atropurpurea* shells as a precursor. The experimental characterizations indicated the successful N-doping of the CQDs on the active materials. The electrochemical performances of the electrodes revealed that NCQDs synthesized from the KOH activation agent achieved a commercial-like supercapacitor electrode compared to NCQDs prepared from H_2_SO_4_ and NaOH activation agents. Moreover, under the KOH activation condition of *Callerya Atropurpurea* shells, the NCQDs-3 electrode achieved a coulombic efficiency of 98% and capacitive retention of 99% after 1000 GCD cycles. This was attributed to the facilitation of profuse active sites for electrolyte diffusion, promoting better electron transport and rapid reaction kinetics, making them suitable and potential electrode materials for energy storage applications.

## Data Availability

The data set for this work is available on reasonable request. Any enquiry about data enquiry should be addressed to Abdulrahman Oyekanmi Adeleke.
